# Identification of key genes in gout and atherosclerosis and construction of molecular regulatory networks

**DOI:** 10.3389/fcvm.2024.1471633

**Published:** 2024-11-29

**Authors:** Gong Qing, Zujun Yuan

**Affiliations:** Gastroenterology Department, The People’s Hospital of Chongqing Liangping District, Chongqing, China

**Keywords:** gout, atherosclerosis, regulatory network, transcription factors, miRNA

## Abstract

**Background:**

Gout is a type of chronic inflammatory disease linked to the accumulation of monosodium urate crystals, leading to arthritis. Studies have shown that patients with gout are more likely to develop atherosclerosis, but the specific mechanisms involved remain unknown. The purpose of the research was to explore the key molecules and potential mechanisms between gout and atherosclerosis.

**Methods:**

Gene expression profiles for gout as well as atherosclerosis were obtained from the Gene Expression Omnibus (GEO) database, then differential analysis was utilized to identify common differentially expressed genes (DEGs) between the two diseases. The analysis of functional enrichment was conducted to investigate the biological processes that the DEGs might be involved in. The Cytoscape software was utilized to develop a protein–protein interaction (PPI) network as well as identify hub genes, while LASSO analysis was employed to select key genes. The TRRUST database was utilized to forecast transcription factors (TFs), and the miRTarBase database was utilized to forecast miRNAs.

**Results:**

Four key genes, CCL3, TNF, CCR2, and CCR5, were identified. The receiver operating characteristic (ROC) curves showed that the areas under ROC curve (AUC) for these four key genes in both gout and atherosclerosis were greater than 0.9. The analysis of functional enrichment revealed that the DEGs were primarily involved in “regulation of T-cell activation”, “chemokine signaling pathway”, and other biological processes. The TRRUST prediction results indicated that RELA and NFKB1 are common regulatory transcription factors for CCR2, CCR5, CCL3, and TNF. The miRTarBase prediction results showed that hsa-miR-203a-3p is a common regulatory miRNA for TNF and CCR5.

**Conclusion:**

This study preliminarily explored the potential key molecules and mechanisms between gout and atherosclerosis. These findings provide new insights for further research into identifying potential biomarkers and clinical treatment strategies for these two diseases.

## Introduction

Gout is a metabolic illness caused by a disorder in purine metabolism, leading to elevated blood uric acid levels as well as the formation and accumulation of monosodium urate crystals within joints as well as other tissues (1). Its hallmark symptom is acute arthritis, a type of inflammatory response that not only causes severe pain but can also lead to long-term joint damage and dysfunction, significantly affecting patients' quality of life. In recent years, with changes in lifestyle, the incidence of gout has been on the rise, becoming one of the global public health issues (2). Research shows that approximately 41.2 million adults worldwide suffer from gout, more than twice the number of those with rheumatoid arthritis (3, 4). In the United States alone, the number of adults with gout is as high as 9.2 million, accounting for about 3.9% of the total adult population (5).

In recent decades, a substantial amount of research has indicated a close association between gout or hyperuricemia and cardiovascular diseases (6–8). Notably, the incidence of cardiovascular diseases in gout patients is significantly higher compared to the general population, suggesting the potential existence of common pathophysiological mechanisms between the two conditions. Atherosclerosis is a type of chronic cardiovascular disease caused by the buildup of lipids as well as fibrous components in the arterial walls, leading to reduced elasticity, narrowing of the vessel lumen, and potentially resulting in heart and cerebrovascular diseases, as well as disabling peripheral artery disease (9). Hyperuricemia increases the risk of cardiovascular disease via regulating inflammatory responses, oxidative stress, and endothelial dysfunction (10). Uric acid can activate endothelial cells, promoting the release of inflammatory factors as well as increasing oxidative stress, thereby damaging endothelial function and accelerating the process of atherosclerosis (11). A study found that compared with healthy participants, gout patients have shorter telomeres, and the telomere length is associated with the frequency of gout attacks as well as cardiovascular diseases (12). This could be a major factor for the increased risk of atherosclerosis in gout patients.

Although numerous studies support the association between gout and atherosclerosis, the specific molecular mechanisms between them remain unclear. This gap in understanding limits our ability to prevent and treat cardiovascular diseases related to gout and hyperuricemia. In particular, a lack of deep understanding of the common molecular pathways and key regulatory factors between the two diseases is crucial for developing new therapeutic methods and intervention strategies. Therefore, exploring the connection between gout and atherosclerosis and clarifying their common molecular mechanisms are not only significant for understanding the intrinsic link between these two diseases but also provide new ideas for developing targeted treatment strategies.

**Figure 1:**
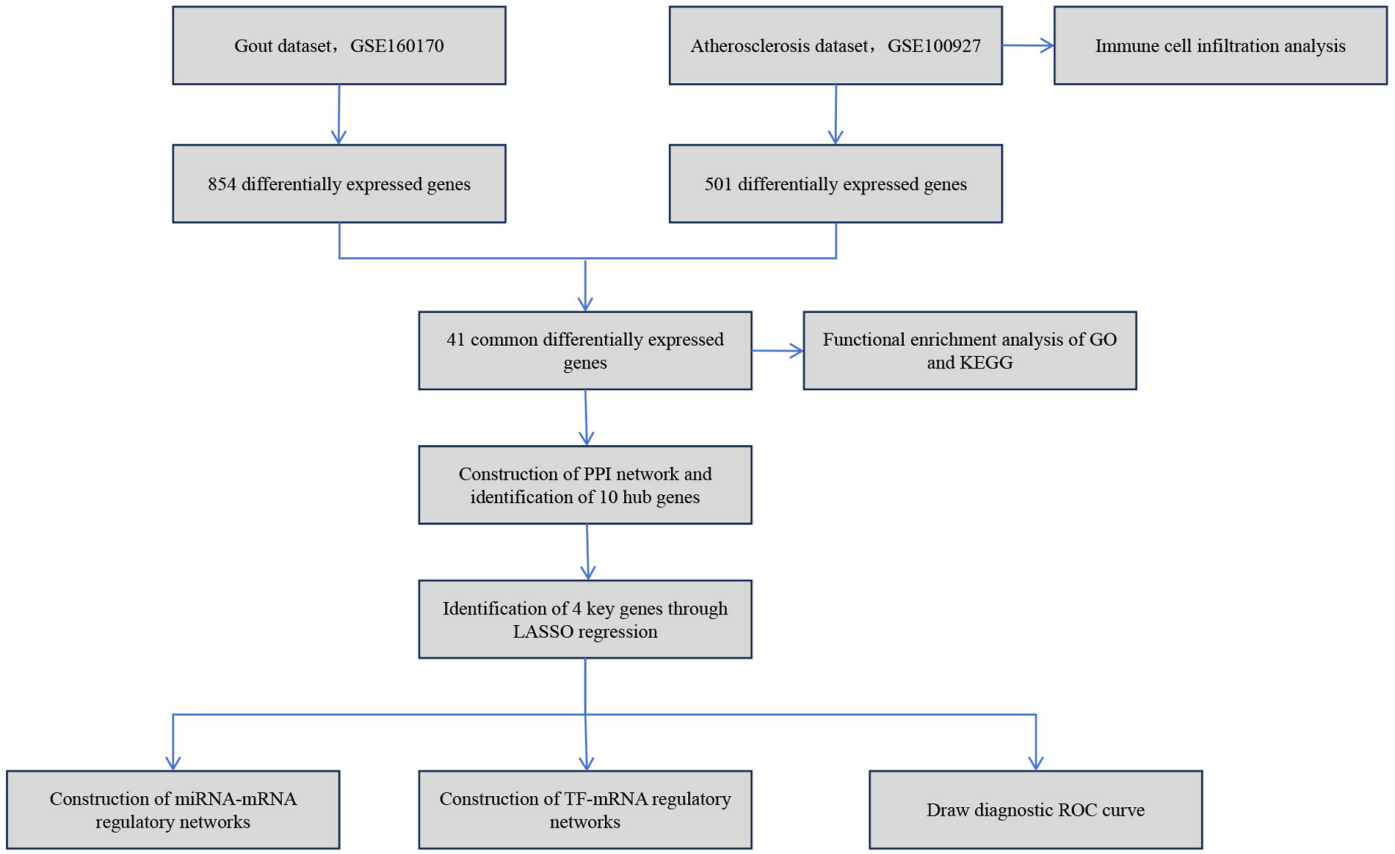
The simplified analysis workflow of this study.

Thus, this study is dedicated to thoroughly analyzing the relationship between gout and atherosclerosis, with a focus on revealing the common molecular mechanisms of the two diseases. By comprehensively applying various bioinformatics techniques, this study successfully identified the key molecules and their signaling pathways commonly related to both gout and atherosclerosis. Moreover, the study constructed the regulatory networks of these key molecules involving transcription factors and miRNAs. The findings not only enrich our understanding of the relationship between gout and atherosclerosis but also offer a new theoretical basis and research direction for future exploration of the interaction mechanisms between these two diseases. The simplified analysis workflow of this study is illustrated in Figure 1.

## Materials and methods

### Source of original data

RNA-seq datasets for gout as well as atherosclerosis were retrieved and obtained from the Gene Expression Omnibus (GEO) database. The gout dataset (GSE160170) consisted of samples from 6 patients with primary gout as well as 6 healthy control participants. The atherosclerosis dataset (GSE100927) was composed of samples from 69 patients with peripheral artery atherosclerosis and 35 control tissues.

### Selection of differentially expressed genes

Differential expression analysis between samples from gout and atherosclerosis and their respective control groups was conducted utilizing the “limma” package in R (13). The criteria for selection were |Log2FC|>1 as well as p.adj <0.05, in order to identify significantly differentially expressed genes (DEGs). Volcano plots were then constructed utilizing the “ggplot2” package in R in order to visualize the DEGs (Supplementary Table S1). The “VennDiagram” package in R was applied to identify common DEGs among gout and atherosclerosis (Supplementary Table S2). Finally, a heatmap of the DEGs was visualized utilizing the “ComplexHeatmap” package in R (14).

### Functional enrichment analysis and construction of the PPI network

The common DEGs among gout and atherosclerosis were assessed to Gene Ontology (GO) as well as Kyoto Encyclopedia of Genes and Genomes (KEGG) analyses utilizing the “clusterProfiler” R package (15) (Supplementary Table S3). The STRING (https://cn.string-db.org/) database was utilized to obtain protein information and protein-protein interaction (PPI) network information (Supplementary Table S4), which was then displayed via Cytoscape software.

### Identification of key genes

The common DEGs were further analyzed utilizing the CytoHubba plugin in Cytoscape software, employing the Degree approach to identify the top ten hub genes (Supplementary Table S5). To further identify key genes in gout and atherosclerosis, these ten hub genes were performed to LASSO analysis utilizing the “glmnet” package in R (Supplementary Table S6). The ROC curves for the key genes were then shown utilizing the “pROC” package in R (Supplementary Table S7).

### Immune cell infiltration analysis

In order to evaluate the infiltration of immune cells within atherosclerosis tissue samples, the signature matrix gene expression profiles for 22 categories of immune cells provided by the CIBERSORTx website (https://cibersortx.stanford.edu/) were employed (Supplementary Table S8). Based on the core algorithm provided in the “CIBERSORT” R package, the infiltration level of immune cells in each sample was calculated (16, 17).

### Construction of the molecular regulatory network

The TRRUST database (https://www.grnpedia.org/trrust/) was utilized to predict transcription factors (TFs) that have regulatory relationships with target genes. The miRTarBase database (https://mirtarbase.cuhk.edu.cn/) was utilized to predict miRNAs that have regulatory relationships with target genes. Finally, Cytoscape software was utilized to construct the TF-mRNA and miRNA-mRNA regulatory networks (Supplementary Table S9).

### Statistical methods

The present research's statistical analysis was performed utilizing R software (version 4.2.1). For continuous variables, the *t*-test (for two groups) either one-way ANOVA (for multiple groups) was applied if the data followed a normal distribution. For categorical variables and continuous variables not following a normal distribution, the Wilcoxon test was used. All statistical analyses were considered statistically significant with a *P*-value <0.05.

## Results

### Identification of common DEGs between gout and atherosclerosis

**Figure 2:**
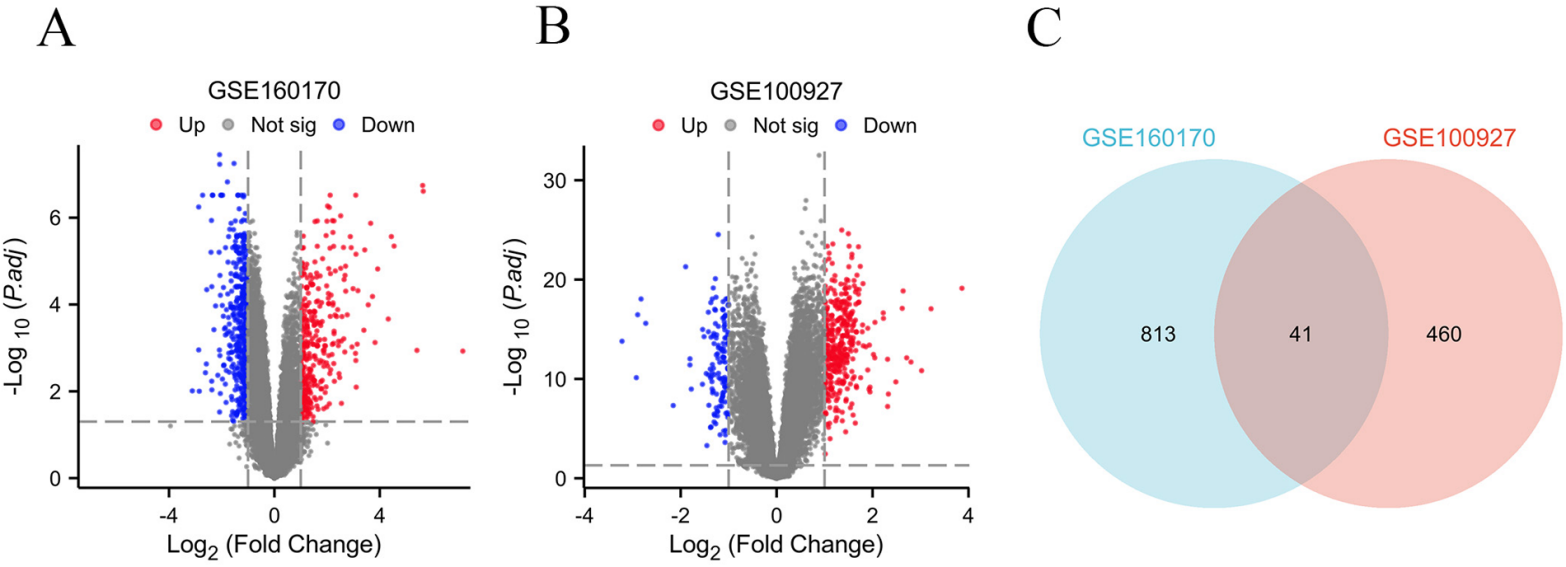
Identification of common DEGs between gout and atherosclerosis. **(A)** Volcano plot of DEGs in gout; **(B)** Volcanic plot of DEGs in atherosclerosis; **(C)** Venn diagram of DEGs in gout and atherosclerosis.

**Figure 3:**
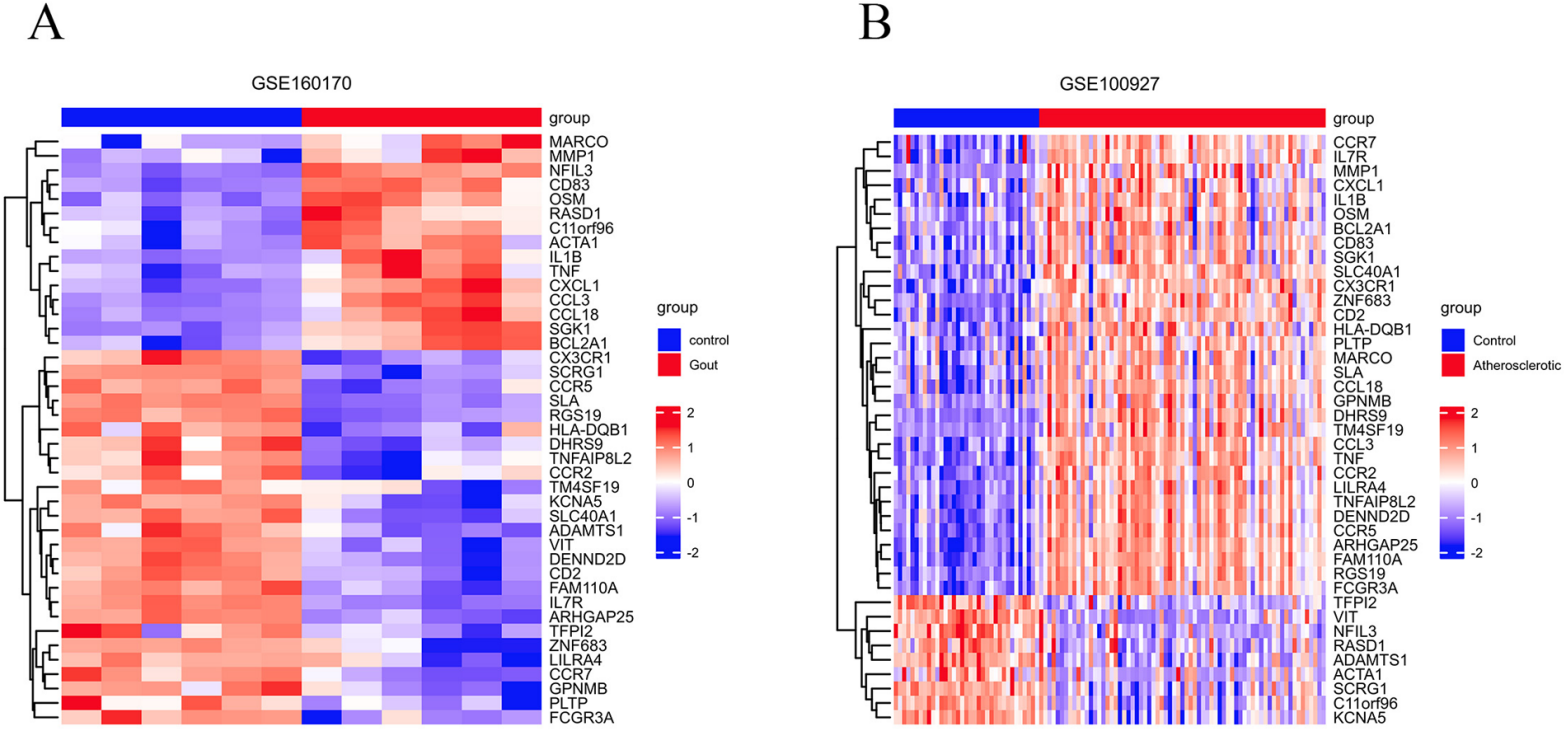
The 41 common DEGs between gout and atherosclerosis. **(A)** Heatmap of common DEGs in gout; **(B)** heatmap of common DEGs in atherosclerosis.

Based on the criteria for differentially expressed genes (|Log2FC|>1 as well as p.adj <0.05), 854 differentially expressed genes (DEGs) were identified in the gout samples (Figure 2A), as well as 501 DEGs were identified in the atherosclerosis samples (Figure 2B), both presented in the form of volcano plots. Subsequently, an intersection of the DEGs among gout and atherosclerosis yielded 41 common DEGs, displayed in a Venn diagram (Figure 2C). Lastly, the expression patterns of these 41 common DEGs in samples from both gout and atherosclerosis were showcased in heatmaps (Figures 3A,B).

### Functional enrichment analysis

**Figure 4:**
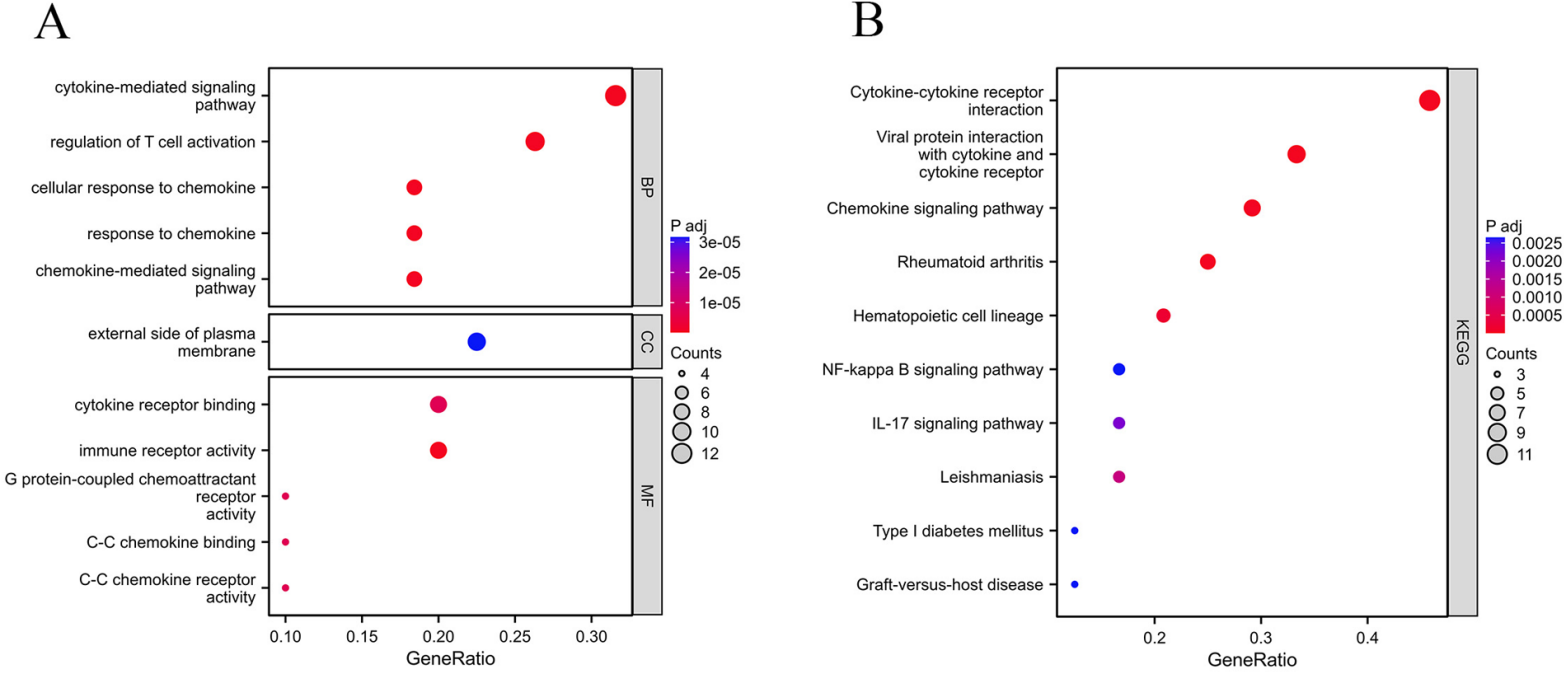
Functional enrichment analysis. **(A)** GO enrichment analysis; **(B)** KEGG enrichment analysis.

In order to investigate the biological roles of the 41 common DEGs between gout and atherosclerosis, GO as well as KEGG enrichment analyses were performed. The findings revealed that the common DEGs were primarily involved in “regulation of T cell activation”, “chemokine-mediated signaling pathway”, “cytokine-mediated signaling pathway”, as well as “cytokine receptor binding” (Figure 4A). The KEGG analysis revealed that these genes were significantly involved in pathways like “cytokine-cytokine receptor interaction”, “chemokine signaling pathway”, and “interaction between viral proteins and cytokines and cytokine receptors” (Figure 4B).

### PPI network and key gene selection

**Figure 5:**
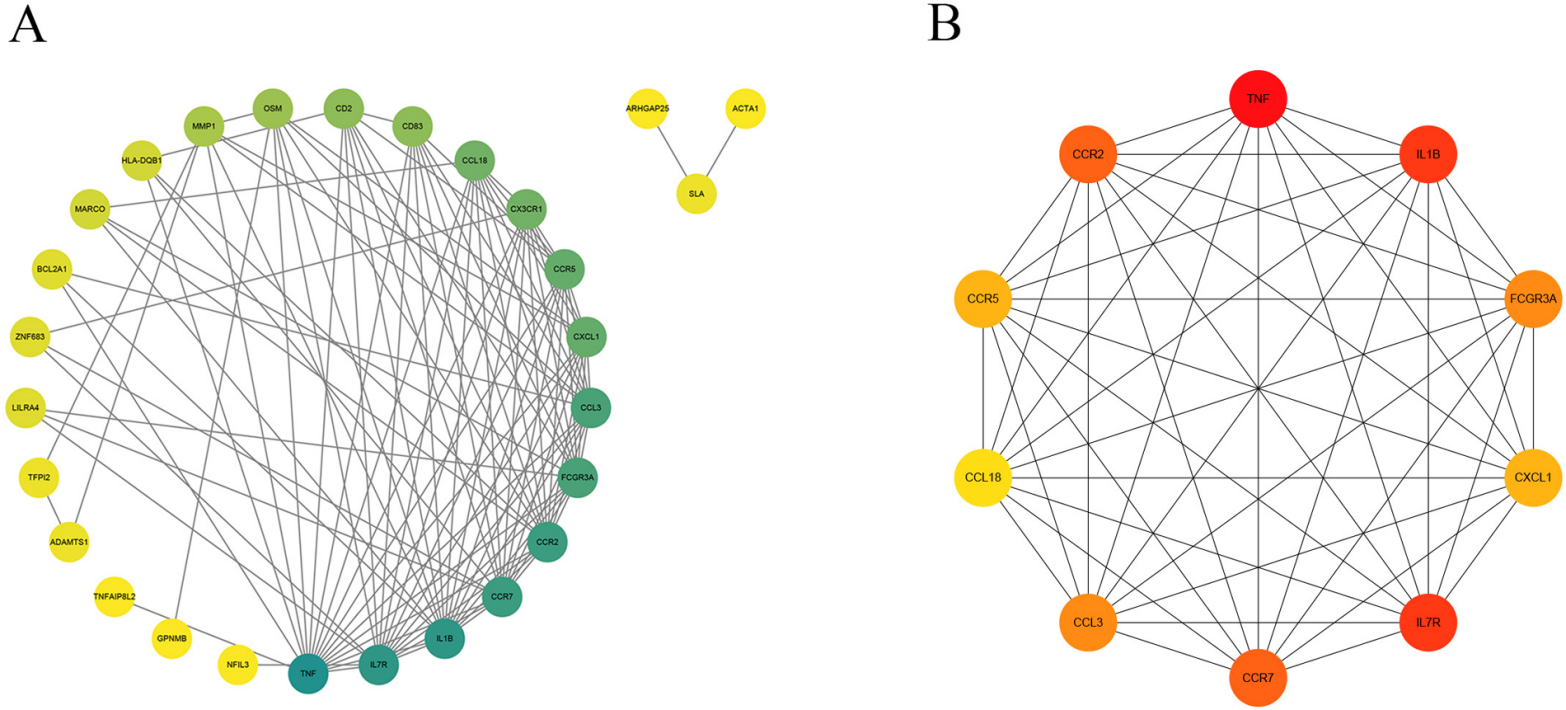
PPI network and top 10 hub genes. **(A)** The PPI network of 41 common DEGs; **(B)** identification of 10 hub genes.

**Figure 6:**
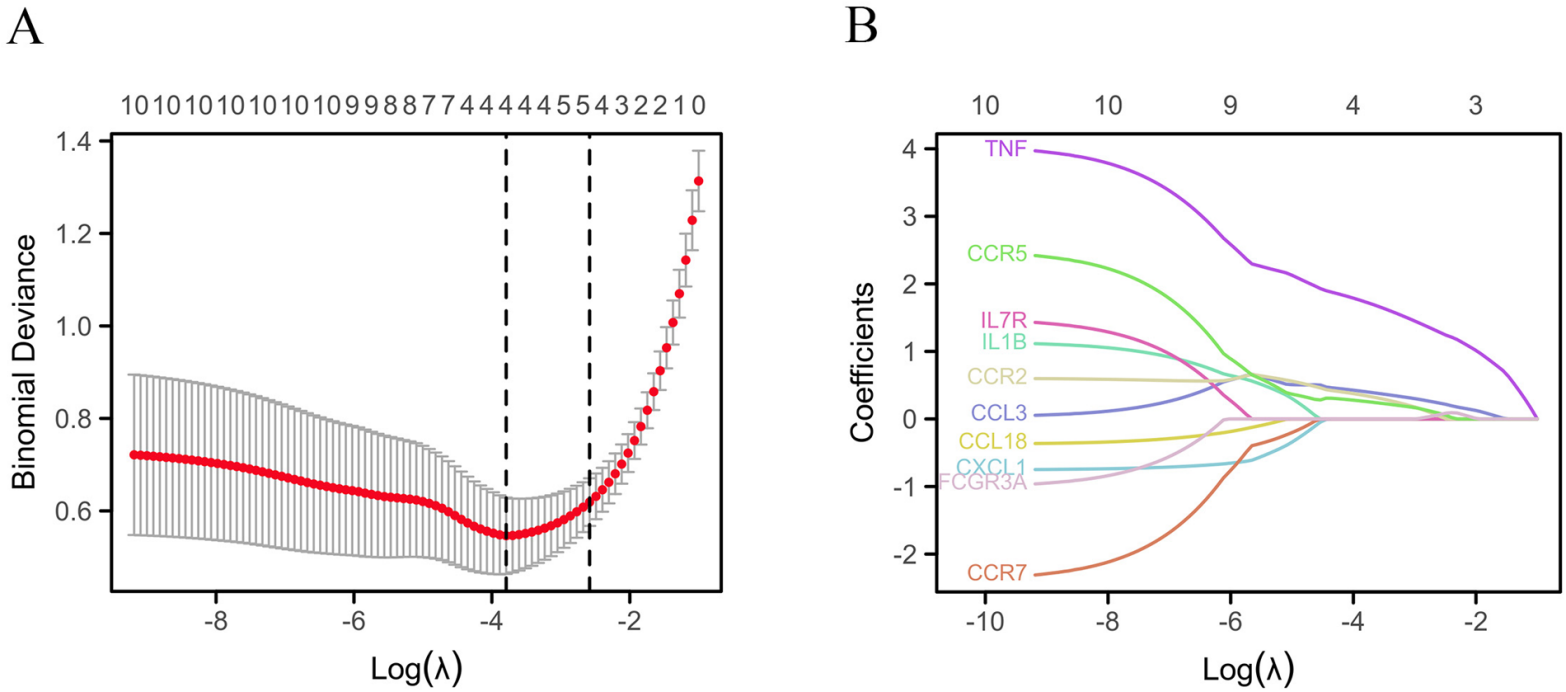
Identification of key genes. **(A)** Lasso cross validation diagram; **(B)** Lasso coefficient analysis diagram.

**Figure 7:**
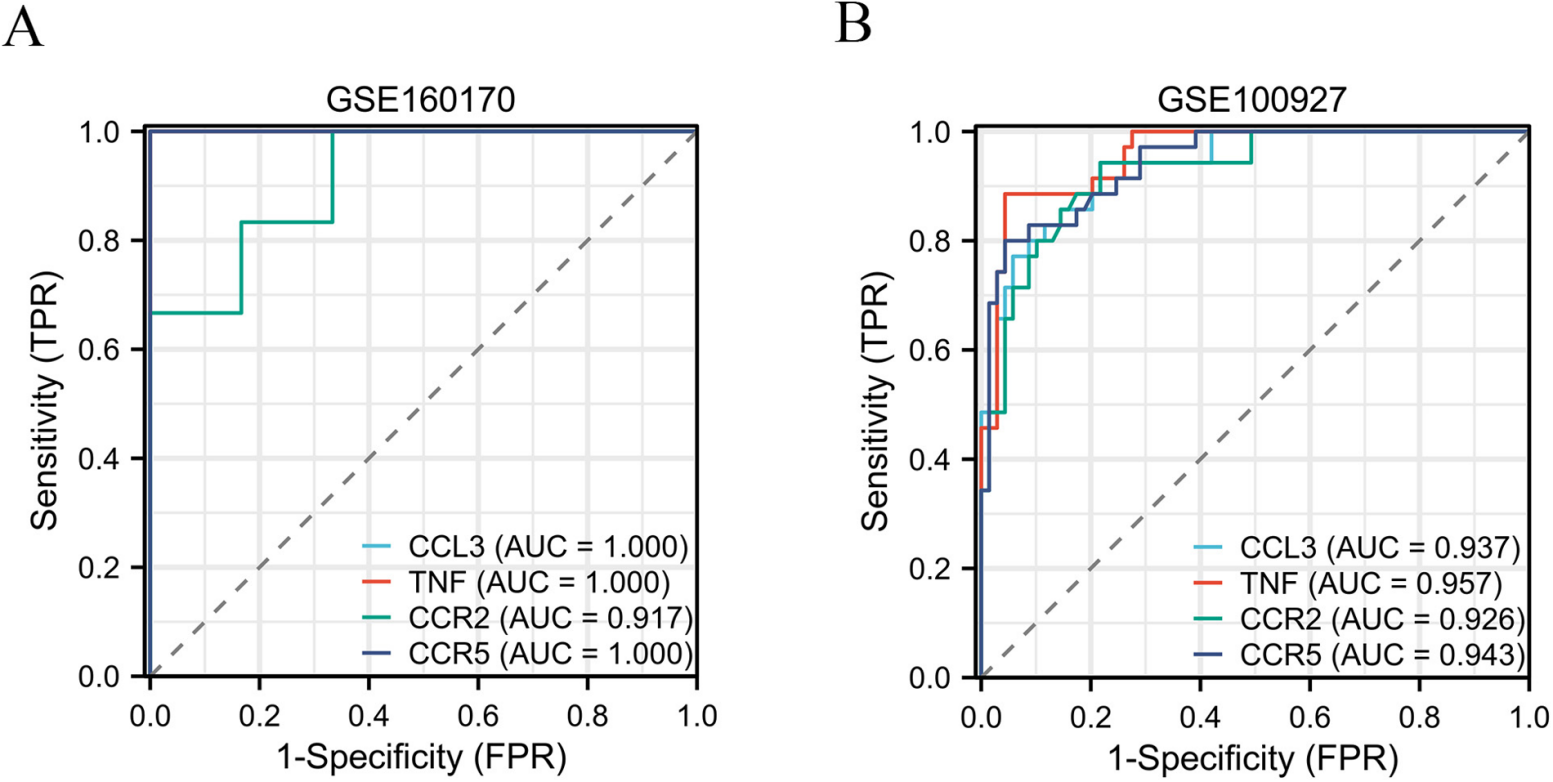
The ROC curves were utilized to assess the diagnostic value of these key genes in gout **(A)** and atherosclerosis **(B)**.

The STRING database was applied to collect PPI network information among the common DEGs among gout and atherosclerosis, which was visualized using Cytoscape software (Figure 5A). Subsequently, the top 10 hub genes, including CCL3, CCL18, IL1B, CXCL1, TNF, CCR7, IL7R, CCR2, CCR5, and FCGR3A, were identified using the Degree method in the CytoHubba plugin of Cytoscape software (Figure 5B). Further selection of key genes through LASSO analysis method yielded four key genes: CCR2, CCR5, CCL3, and TNF (Figures 6A,B). The λ values in LASSO analysis were detailed in Supplementary Table S6. The ROC curves were utilized to assess the diagnostic value of these key genes in gout and atherosclerosis, with all four key genes showing the areas under the curve greater than 0.9, indicating their significant diagnostic value in both diseases (Figures 7A,B).

### Construction of miRNA-mRNA and TF-mRNA regulatory networks

**Figure 8:**
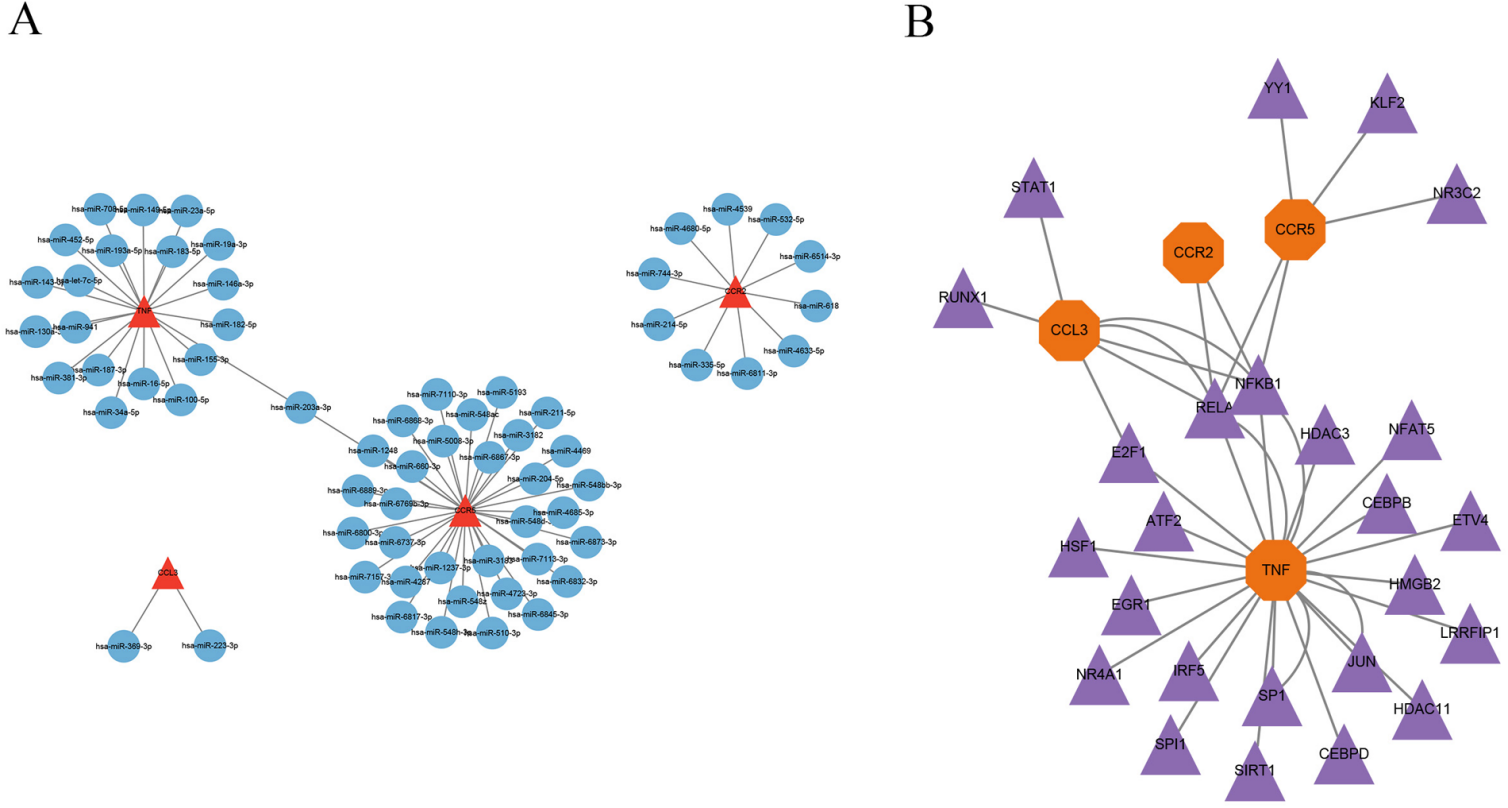
Construction of miRNA-mRNA and TF-mRNA regulatory networks. **(A)** miRNA-mRNA regulatory network; **(B)** TF-mRNA regulatory network.

The miRTarBase database was employed to forecast miRNAs that regulate the target genes, and the miRNA-mRNA regulatory network was created via Cytoscape software. The results indicated that hsa-miR-203a-3p is a common regulatory miRNA for the key genes TNF and CCR5 (Figure 8A). The predicted transcription factors for the four key genes were obtained from the TRRUST database, and the TF-mRNA network was created via Cytoscape software, showing RELA and NFKB1 as common regulatory transcription factors for CCR2, CCR5, CCL3, and TNF, while E2F1 was identified as a common regulatory transcription factor for CCL3 and TNF (Figure 8B).

### Immune cell infiltration analysis

**Figure 9:**
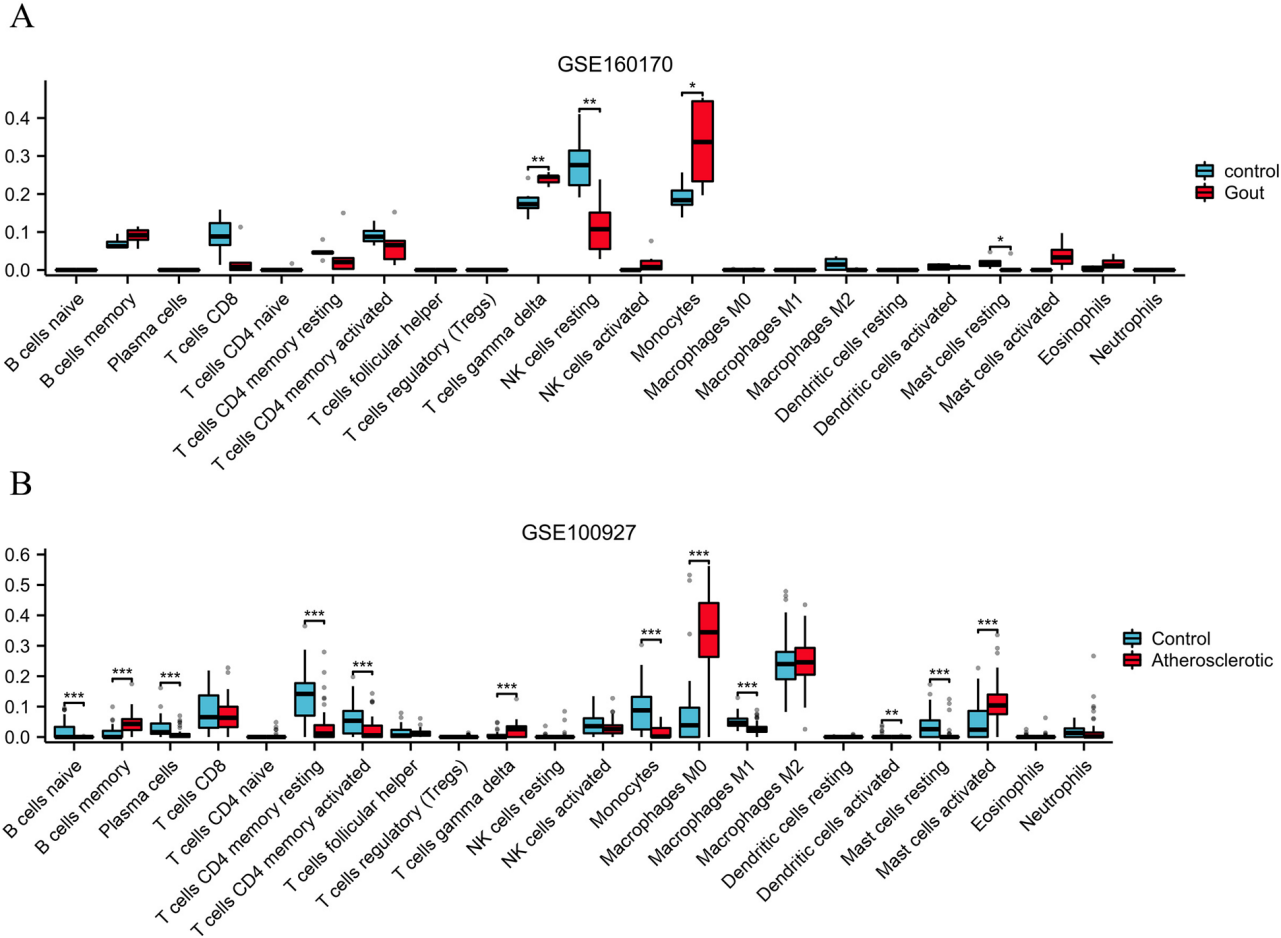
Analysis of immune cell infiltration in gout **(A)** and atherosclerotic **(B)** tissue samples.

To assess the infiltration level of immune cells in gout and atherosclerotic tissue samples, the “CIBERSORT” R package's core algorithm was used to calculate the infiltration level of various immune cells in each sample. The results demonstrated significant differences in the infiltration level of T cell gamma delta, NK cell resting, and monocytes between gout tissue samples and control samples (Figure 9A). Similarly, there were significant differences in the infiltration level of various immune cells between atherosclerosis tissue samples and control samples, including B cells memory, T cells gamma delta, Monocells M0 and Mast cells activated (Figure 9B).

## Discussion

This study aimed to delve into the potential connection between gout and atherosclerosis, particularly from a molecular mechanism perspective. Gout is an inflammatory disease associated with uric acid metabolism abnormality, caused by the accumulation of monosodium urate crystals within joints as well as soft tissues, leading to recurrent episodes of acute arthritis. Atherosclerosis, on the other hand, is a chronic cardiovascular illness caused by the build-up of lipids as well as fibrous tissues in the wall of arteries, resulting in reduced vascular elasticity and lumen narrowing. Although the link between the two has been somewhat confirmed by related studies, the specific molecular mechanisms and biological processes involved remain unclear. By employing bioinformatics methods, we conducted a comprehensive analysis of gene expression profiles of gout as well as atherosclerosis through public databases and successfully identified 41 common DEGs, mainly enriched in key biological processes such as chemokine signaling pathways and regulation of T-cell activation. Through LASSO analysis, we ultimately identified CCL3, TNF, CCR2, and CCR5 as key genes, which showed high diagnostic value in gout and atherosclerosis through ROC curve analysis. Additionally, we constructed miRNA-mRNA and TF-mRNA regulatory networks, finding that factors such as hsa-miR-203a-3p, RELA, and NFKB1 play significant roles in regulating these key genes. Immune cell infiltration analysis further revealed changes in specific immune cell types in tissues of gout and atherosclerosis.

The identification of 41 common DEGS in this study suggests potential molecular regulation and signaling pathways shared between gout and atherosclerosis. These genes were primarily enriched in “regulation of T-cell activation”, “chemokine signaling pathway”, as well as “cytokine-mediated signaling pathway”, which are significant for understanding the molecular link between gout and atherosclerosis. “Regulation of T-cell activation” is a core process in the immune system, involving the initiation and regulation of immune responses. In gout, abnormal activation of T-cells may lead to an excessive response to urate crystals, triggering inflammation (18, 19). Similarly, the development of atherosclerosis is also associated with T-cell mediated inflammatory responses, especially in the damage to arterial endothelium and plaque formation processes (20). Thus, the role of these common differentially expressed genes in regulating T-cell activation may be a key link connecting gout and atherosclerosis. The “chemokine signaling pathway” is essential to regulate the migration as well as localization of immune cells. During a gout attack, the release of chemokines promotes the migration of immune cells to the inflammation site to participate in the inflammatory response (21). Likewise, in the progression of atherosclerosis, chemokines also involve the accumulation of immune cells, particularly macrophages, in the vascular wall, which is crucial for plaque formation (22, 23). Therefore, the role of common DEGs in the chemokine signaling pathway emphasizes the common mechanism of immune cells in both diseases. The “cytokine-mediated signaling pathway” is another key pathway regulating intercellular communication. In the pathological processes of both gout and atherosclerosis, cytokines, such as tumor necrosis factor alpha (TNF-α) as well as interleukins (ILs), play central roles in inflammation and immune regulation (24–27). The enrichment of common DEGs in this process may reflect a common pathway by which cytokines regulate the pathological states of both diseases.

In this study, through comprehensive analysis methods, four key genes—CCL3, TNF, CCR2, and CCR5—were identified, having significant impacts on the pathophysiological mechanisms of gout and atherosclerosis. These genes not only exhibit significant differential expression in both diseases but also demonstrate high diagnostic value through ROC curve analysis, suggesting their core roles in disease progression. CCL3, a chemokine, attracts immune cells such as monocytes and T cells to inflammation sites, with CCR5 acting as its receptor involved in the migration and activation of immune cells (28–30). This process plays a key role in the inflammatory response during gout attacks and may also contribute to the formation and development of atherosclerotic plaques by promoting immune cell accumulation in the vascular wall. TNF, a critical inflammatory factor, was proven to possess significant functions in the biological mechanisms of both gout and atherosclerosis (31–33). TNF can promote the production of inflammatory cytokines, exacerbate inflammatory responses, and participate in regulating the function to vascular endothelial cells, affecting the progression of atherosclerosis (34). Thus, TNF may be an important link connecting the pathophysiological mechanisms of gout and atherosclerosis. CCR2, another crucial chemokine receptor, primarily regulates the migration of monocytes (35, 36). In atherosclerosis, the activation of CCR2 promotes monocyte migration to the vascular wall and their transformation into macrophages, participating in plaque formation (37). Similarly, CCR2 may also play an important role in the inflammatory response of gout via regulating the migration and activation of immune cells. The identification of these key genes not only deepens our understanding about the pathophysiological mechanisms of gout and atherosclerosis but also identifies new potential targets for future therapeutic strategies.

By constructing miRNA-mRNA and TF-mRNA regulatory networks, we further explored the upstream regulatory mechanisms of key genes in the processes of gout and atherosclerosis. The discovery of factors such as hsa-miR-203a-3p, RELA, and NFKB1 provides new insights into the molecular mechanisms and therapeutic potential of these illnesses. miRNAs, a category of non-coding RNAs, regulate gene expression through attachment to the 3′ untranslated region (3′UTR) of their target mRNAs (38). In this study, hsa-miR-203a-3p was predicted to regulate TNF and CCR5, suggesting that miRNAs might affect the progression of gout as well as atherosclerosis by modulating the expression of these key inflammatory and immune-related genes. Transcription factors, proteins within cells responsible for initiating or inhibiting gene transcription, thus controlling gene expression. In our study, RELA and NFKB1, as transcription factors, were discovered to be associated with the expression regulation of key genes such as CCR2, CCR5, CCL3, and TNF. The NF-κB signaling pathway, regulated by RELA and NFKB1, is one of the core pathways modulating inflammatory responses and immune reactions (39, 40). Its activation plays an important role in both the acute attacks of gout and the formation of atherosclerotic plaques, revealing it as a potential molecular mechanism linking the two diseases. The discovery of these regulatory networks not only enhances our understanding of the comorbidity mechanisms between gout and atherosclerosis but also provides new directions for future therapeutic research. Therapeutic strategies targeting specific miRNAs or transcription factors, such as using miRNA mimics or inhibitors, as well as developing small molecule inhibitors against the NF-κB signaling pathway, could emerge as new approaches for treating gout with concurrent atherosclerosis.

Immune cell infiltration analysis has shown significant changes in various immune cells in tissue samples from gout and atherosclerosis, which is of great importance for understanding the pathophysiological mechanisms of these two diseases. Gout is a chronic inflammatory disease triggered by the deposition of urate crystals, while atherosclerosis is characterized by lipid deposition and inflammatory responses within the blood vessels (41, 42). In the progression of both diseases, the infiltration and activation status of immune cells play a crucial role. In gout, the immune response to urate crystals initiates inflammation, resulting in joint pain and swelling. Notably, activated macrophages contribute to this process by releasing multiple inflammatory mediators, such as tumor necrosis factor-alpha (TNF-α) and interleukin-1 beta (IL-1β), which exacerbate the local inflammatory response (43, 44). These mediators not only increase the number of leukocytes at the affected joint but also trigger a more widespread systemic inflammatory response, potentially correlating with the risk and prognosis of cardiovascular diseases. Meanwhile, the formation of atherosclerosis is inextricably linked to immune cell infiltration. Research indicates that various immune cells, including T cells and macrophages, play key roles in the formation and progression of atherosclerotic plaques (45, 46). T cells infiltrating and activating in the arterial wall promote localized inflammation and increase the secretion of inflammatory mediators, further driving plaque formation and instability (47). Additionally, macrophages not only are crucial in the inflammatory response but also participate in lipid uptake and metabolism. When macrophages engulf excessive lipids, they may transform into foam cells, which promote the progression of atherosclerosis (48). Furthermore, the pathological process of atherosclerosis also involves the participation of other immune cells such as B cells and mast cells, which are important in regulating adaptive immune responses and maintaining the stability of the inflammatory microenvironment (49, 50). These immune cells interact through various mechanisms, collectively facilitating the occurrence and development of atherosclerosis. In this process, the degree of immune cell infiltration and activation status provides important clues for understanding the common pathological mechanisms of gout and atherosclerosis. Combining current research findings, the characteristics of immune cell infiltration in gout and atherosclerosis suggest that these two diseases may have interrelated pathogenic mechanisms. The enhancement of inflammatory responses may not only be a product of local joint damage but could also impact cardiovascular health through systemic effects. Therefore, identifying methods to regulate immune cell infiltration carries significant clinical importance.

Despite the valuable insights into the molecular foundations of gout as well as atherosclerosis provided by this study, there are certain limitations. First of all, this work relies on gene expression data through public databases, which may have limited sample sizes and come from specific populations or geographic locations, potentially limiting the generalizability of the results. Larger-scale, diverse populations, and multicenter sample collections could help enhance the representativeness and reliability of the findings. Second, although bioinformatics tools and methods can help identify potential biomarkers and disease-related pathways, these analyses depend on existing algorithms and databases, so new, unstudied mechanisms might be overlooked, and the importance of known pathways might be overstated. Lastly, this study primarily relies on bioinformatics analysis and lacks experimental data to validate key findings, such as the functions of key genes, the regulatory roles of miRNAs, and transcription factors. Experimental validation is an important step to confirm the biological significance of these bioinformatics predictions.

## Conclusion

This study investigated the molecular link between gout as well as atherosclerosis. Via the analysis of gene expression profiles of patients with gout and atherosclerosis, we successfully identified 41 DEGs, primarily involved in crucial processes in biology like “regulation of T-cell activation” and “chemokine signaling pathways”, suggesting that gout and atherosclerosis may be connected through these common molecular pathways. Further bioinformatics analysis highlighted the significant roles of CCL3, TNF, CCR2, and CCR5 in both diseases. Moreover, our study constructed miRNA-mRNA and TF-mRNA regulatory networks, finding that regulators such as hsa-miR-203a-3p, RELA, and NFKB1 play core roles in this network, further supporting the complex molecular interactions between gout and atherosclerosis. The results of immune cell infiltration analysis revealed significant changes in specific types of immune cells in these diseases, emphasizing the importance of immune regulation in disease progression. In conclusion, the results of the present study provide a novel theoretical basis for the comorbidity mechanism of gout as well as atherosclerosis, pointing the way for future basic and clinical research. By further revealing the specific mechanisms of these key genes and regulators, we seek to develop novel strategies for preventing, diagnosing, as well as treating these illnesses, thereby improving patients’ health and quality of life.

## Data Availability

The original contributions presented in the study are publicly available. This data can be found here from the Gene Expression Omnibus (GEO) database: https://www.ncbi.nlm.nih.gov/geo/ accession numbers (GSE160170) and (GSE100927).
